# Frequencies, Modalities, Doses and Duration of Computerized Prescriptions for Sedative, Analgesic, Anesthetic and Paralytic Drugs in Neonates Requiring Intensive Care: A Prospective Pharmacoepidemiologic Cohort Study in 30 French NICUs From 2014 to 2020

**DOI:** 10.3389/fphar.2022.939869

**Published:** 2022-07-18

**Authors:** Manon Tauzin, Béatrice Gouyon, Déborah Hirt, Ricardo Carbajal, Jean-Bernard Gouyon, Anne-Claire Brunet, Matthieu Ortala, Seydou Goro, Camille Jung, Xavier Durrmeyer

**Affiliations:** ^1^ Neonatal Intensive Care Unit, CHI Créteil, Créteil, France; ^2^ Centre d’Etudes Périnatales de L’Océan Indien (CEPOI, EA7388), Université de La Réunion, Saint Pierre, France; ^3^ Pharmacology Department, Hôpital Cochin APHP, Paris, France; ^4^ Pediatric Emergency Department, Assistance Publique-Hôpitaux de Paris, Hôpital Armand Trousseau- Sorbonne Université, Paris, France; ^5^ Institut National de La Santé et de La Recherche Médicale UMR1153, Paris, France; ^6^ Kaduceo SAS, Toulouse, France; ^7^ Clinical Research Center, CHI Créteil, Créteil, France; ^8^ Faculté de Médecine de Créteil, IMRB, GRC CARMAS, Université Paris Est Créteil, Créteil, France

**Keywords:** neonates, analgesics, sedatives, pain, pharmacoepidemiology, doses

## Abstract

**Objectives:** No consensus exists about the doses of analgesics, sedatives, anesthetics, and paralytics used in critically ill neonates. Large-scale, detailed pharmacoepidemiologic studies of prescription practices are a prerequisite to future research. This study aimed to describe the detailed prescriptions of these drug classes in neonates hospitalized in neonatal intensive care units (NICU) from computerized prescription records and to compare prescriptions by gestational age.

**Materials and Methods:** We included all neonates requiring intensive care in 30 French level III units from 2014 through 2020 with a computerized prescription for an analgesic, sedative, anesthetic, or paralytic agent. We described frequencies of prescription, methods of administration, concomitant drug prescriptions, and dosing regimen, and compared them across gestational ages.

**Results:** Among 65,555 neonates, 29,340 (44.8%) were prescribed at least one analgesic (acetaminophen in 37.2% and opioids in 17.8%), sedative (9.8%), anesthetic (8.5%), and/or paralytic agent (1%). Among preterm infants born before 28 weeks, 3,771/4,283 (88.0%) were prescribed at least one of these agents: 69.7% opioids, 41.2% sedatives, 32.5% anesthetics, and 5.8% paralytics. The most frequently prescribed agents were sufentanil (in 10.3% of neonates) and morphine (in 8.0% of neonates) for opioids, midazolam (9.3%) for sedatives, ketamine (5.7%) and propofol (3.3%) for anesthetics. In most neonates, opioids and sedatives were prescribed as continuous infusion, whereas anesthetics were prescribed as single doses. Opioids, sedatives and paralytics were mostly prescribed in association with another agent. Doses varied significantly by gestational age but within a limited range. Gestational age was inversely related to the frequency, cumulative dose and duration of prescriptions. For example, morphine prescriptions showed median (IQR) cumulative doses of 2601 (848–6750) vs. 934 (434–2679) µg/kg and median (IQR) durations of 7 (3–15) vs. 3 (2–5) days in infants born <28 vs. ≥ 37 weeks of gestation, respectively (*p*-value<0.001).

**Conclusion:** The prescriptions of analgesic, sedative, anesthetic, or paralytic agent were frequent and often combined in the NICU. Lower gestational age was associated with higher frequencies, longer durations and higher cumulative doses of these prescriptions. Dose-finding studies to determine individualized dosing regimens and studies on long-term neurodevelopmental outcome according to received cumulative doses are required.

## Introduction

Neonates hospitalized in neonatal intensive care units (NICUs) are routinely exposed to acute, prolonged, and/or repetitive pain or stress during procedures or situations such as mechanical ventilation, surgery, endotracheal intubation, aspiration, and punctures, with the highest number of painful procedures among the most premature neonates (Anand, 2001; Carbajal et al., 2008; Roofthooft et al., 2014). Neonates, especially those born preterm, are recognized to respond—and even be more sensitive—to pain (Anand and Hickey, 1987; American Academy of Pediatrics, 2000; Anand, 2001). Thus, they frequently require pharmacological treatment to prevent or manage pain, discomfort and stress during hospitalization. Nonetheless, the use of analgesics, sedatives, anesthetics, and paralytics in neonates remains controversial. On the one hand, it is ethically necessary to prevent and relieve pain in this vulnerable population, and evidence shows that repeated and prolonged pain has deleterious consequences on long-term development and behavior (Anand, 1998; Carbajal et al., 2008; Hall and Anand, 2014; McPherson et al., 2020). On the other hand, the potential neurotoxicity of some analgesics, sedatives, and anesthetics and their potential short- and long-term adverse effects must be considered (Loepke, 2010; McPherson and Grunau, 2014). There are currently few recommendations about the best pharmacological agents or the best dose for most of them, for neonates in general and for preterm patients according to their maturation (Committee on fetus and newborn, 2016; Ancora et al., 2019). For most of these drugs, data are insufficient to determine a minimal effective dose that will limit side effects while ensuring the patient’s comfort and/or analgesia.

To develop effective pain management strategies and individualized dosing regimens, we must first understand the pharmacoepidemiology of these medications and how it differs by gestational age (GA).

The main objective of this study was to use computerized records to describe the prescriptions of analgesics, sedatives, anesthetics, and paralytics for neonates hospitalized in NICUs in France and to compare prescriptions and doses by GA.

## Materials and Methods

### Population and Recorded Data

This observational, prospective, multicenter, pharmacoepidemiologic study took place in all French level III NICUs using the Logipren^®^ (Gouyon et al., 2017, 2019; Martin-Mons et al., 2020) computerized physician order-entry system (CPOE). We included all neonates with a postmenstrual age <45 weeks of gestation, hospitalized in a level III NICU, and who received at least one prescription of a sedative, analgesic, anesthetic and/or paralytic (categorized according to the Anatomical Therapeutic Chemical classification (Anatomical therapeutic chemical (ATC) classification. https://www.who.int/medicines/regulation/medicines-safety/toolkit_atc/en/)) (WHO, 2003) between 6 March 2014 and 3 May 2020. Data on antidotes’ prescription (naloxone and flumazenil) were collected.

The study started when CPOE implementation began. The end date was selected from the start of the study. Data were collected prospectively and analysis was conducted retrospectively.

We excluded treatments for patients who received one of the substances for a use other than analgesia/sedation (acetaminophen for patent ductus arteriosus closure and benzodiazepines for seizures).

The Logipren CPOE is associated with a clinical decision support system that has been described previously (Gouyon et al., 2017, 2019). Briefly, with this system, prescriptions are electronically elaborated by the CPOE system and are validated by the medical prescriber based on the indication and patient characteristics. Once the prescriber has chosen an International Nonproprietary Names drug, its indication and the class of GA, the CPOE system retrieves all the necessary data for the prescription and proposes a prescription in accordance with international recommendations. The system provides a complete prescription made from a reference formulary of 450 medications according to their International Nonproprietary Names, based on the French (European) Summary of Product Characteristics for licensed medications (http://agence-prd.ansm.sante.fr/php/ecodex). All electronic prescriptions are automatically stored on local computer servers; data are pseudonymized within each participating center before being sent to the data warehouse monthly. The National Commission for Data Protection and Privacy authorized the use of these prescription data (CNIL: DE-2015-099, DE-2017-410), in accordance with the most recent French regulations on health data research (MR-003).

For this study, we extracted detailed prescription data for each drug: route of administration; doses, including unitary dose/kg and interdose intervals for intermittent administration and single doses; initial dose, maintenance dose (dose/kg/h), and loading dose for continuous administrations; prescription start and end dates; combination of drugs on the same prescription; and patients’ clinical information: gender, GA at birth (weeks), birth weight (g), weight at prescription start and daily weight during the treatment.

### Statistical Analysis

Frequencies of patients with a prescription for each medication were calculated by using the total number of patients with a prescription using the Logipren^®^ CPOE in all NICUs during the study period as the denominator. To describe prolonged or repeated exposition, frequencies of patients with a prescription for 2 or more days (consecutive or not) were also calculated.

We described dosing regimens prescribed for each drug as follows:

- for medications administered continuously: proportion of neonates prescribed a loading dose and this dose value, initial dose (unit/kg/h), cumulative dose (unit/kg) calculated by summing all doses prescribed during the hospitalization, total duration of prescription (days), mean maintenance dose (unit/kg/h), calculated as the cumulative dose divided by the duration of treatment (h).
- for medications administered intermittently: unit dose, cumulative dose (unit/kg) calculated by summing all doses prescribed during the hospitalization and mean daily doses calculated as cumulative dose divided by duration of treatment (d) (unit/kg/d).

For analyses by GA, we divided the population into four groups: 22–27, 28–31, 32–36, and ≥37 weeks. Within each group, we reported and compared previously defined characteristics of prescriptions for the most commonly prescribed medications. We used the nonparametric Kruskal–Wallis test to compare the four groups and then the Wilcoxon signed-rank test to compare groups pairwise, with the Bonferroni method for post hoc analysis. Given the paucity of missing data, they were excluded from the analysis without attempting to replace them. A *p*-value of 0.05 was considered significant. All analyses were performed with R software, version 4.0.5.

## Results

### Population

**FIGURE 1:**
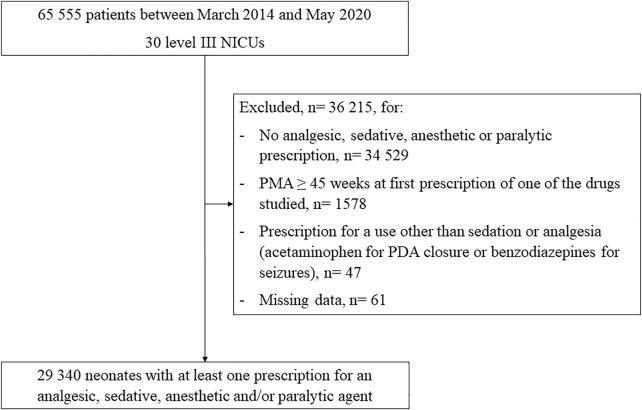
Population flow chart Abbreviations: PMA, post-menstrual age (weeks); PDA, patent ductus arteriosus; NICU, neonatal intensive care unit.

Overall, 65,555 patients had at least one prescription listed on the Logipren CPOE from March 2014 to May 2020 in a level III NICU. Among them, 4,283 (6.5%) infants were born before 28 weeks, 7,877 (12.0%) between 28 and 31 weeks, 22,040 (33.6%) between 32 and 36 weeks, and 31,355 (47.8%) at ≥ 37 weeks. From this initial population, 29,340/65,555 (44.8%) infants had at least one prescription of an analgesic, sedative, anesthetic, and/or paralytic agent (Figure 1). Infants with a prescription of at least one agent had a median (IQR) GA at birth of 35.9 weeks (30.9–39), a median (IQR) birth weight of 2,360 g (1,364–3,190), and 16,816 (57.3%) were boys.

### Prescription Frequencies

Prescription frequencies are described in Table 1. Among the total population (*n* = 65,555), 37.2% had a prescription for acetaminophen and 17.8% for at least one opioid. The most frequently prescribed opioids and derivatives were sufentanil (in 10.3% of neonates) and morphine (in 8.0% of neonates). Sedatives were prescribed for 9.8% of neonates, mainly midazolam (9.3%). Anesthetics were prescribed for 8.5% of neonates, mainly ketamine (5.7%) and propofol (3.3%)—only 2.3% of all neonates had repeated prescriptions of anesthetics for 2 or more days, consecutive or not. Among paralytics, which were prescribed for 1.0% of neonates, atracurium was the most frequently used (0.9%), followed by suxamethonium (0.2%). Antidotes were prescribed to 473 neonates (0.7%). The first prescription of an analgesic, sedative, anesthetic and/or paralytic agent occurred on the first day of life for 13,401/29,340 (45.7%) neonates and during the first week of life for 22,354/29,340 (76.2%) neonates.

**TABLE 1:** Frequencies of patients and units with prescriptions of analgesic, sedative, anesthetic, paralytic, and antidote drugs.

Categories	Drug	Total Patients with a Prescription (%) *n* = 65555^a^	Number of Patients with a Prescription for ≥2 days^b^ (%) *n* = 65555^a^	Number of NICUs (%) *n* = 30
Number of neonates with ≥ 1 analgesic, sedative, paralytic and/or anesthetic prescription, *n* (%)		29,340 (44.8)	27,676 (42.2)	30 (100.0)
Non-opioid analgesics, *n* (%)	Acetaminophen	24,395 (37.2)	21,797 (33.2)	30 (100.0)
Number with ≥ 1 analgesic, sedative, paralytic and/or anesthetic prescription excluding acetaminophen, *n* (%)		13,558 (20.7)	10,224 (15.6)	30 (100.0)
Opioids or derivatives, *n* (%)	Total	11,685 (17.8)	9,723 (14.8)	30 (100.0)
	Sufentanil	6,760 (10.3)	5,600 (8.5)	30 (100.0)
	Morphine	5,262 (8.0)	4,472 (6.8)	30 (100.0)
	Fentanyl	1,142 (1.7)	961 (1.5)	9 (30.0)
	Remifentanil	24 (< 0.1)	8 (< 0.1)	1 (3.3)
	Nalbuphine	1,939 (3.0)	1,151 (1.8)	25 (83.3)
	Methadone	2 (< 0.1)	2 (< 0.1)	1 (3.3)
	Tramadol	1 (< 0.1)	1 (< 0.1)	1 (3.3)
Sedatives, *n* (%)	Total	6,412 (9.8)	5,223 (8.0)	30 (100.0)
*Hypnotics*	Midazolam	6,077 (9.3)	4,976 (7.6)	30 (100.0)
	Hydroxyzine	405 (0.6)	274 (0.4)	13 (43.3)
	Diazepam	332 (0.5)	256 (0.4)	20 (66.7)
*Alpha-2-agonists*	Clonidine	175 (0.3)	166 (0.3)	9 (30.0)
	Dexmedetomidine	41 (0.1)	39 (0.1)	3 (10.0)
Anesthetics, n (%)	Total	5,541 (8.5)	1,496 (2.3)	29 (96.7)
	Ketamine	3,709 (5.7)	993 (1.5)	29 (96.7)
	Propofol	2,152 (3.3)	504 (0.8)	23 (76.7)
	Thiopental	47 (0.1)	14 (< 0.1)	11 (36.7)
Paralytics, *n* (%)	Total	679 (1.0)	297 (0.5)	25 (83.3)
	Atracurium	586 (0.9)	285 (0.4)	22 (73.3)
	Suxamethonium	105 (0.2)	5 (< 0.1)	15 (50.0)
	Rocuronium	24 (< 0.1)	12 (< 0.1)	8 (26.7)
	Vecuronium	8 (< 0.1)	1 (< 0.1)	3 (10.0)
Antidotes, *n* (%)	Total	473 (0.7)	41 (0.1)	26 (86.7)
	Naloxone	460 (0.7)	39 (0.1)	26 (86.7)
	Flumazenil	28 (< 0.1)	2 (< 0.1)	9 (30.0)

NICU, Neonatal intensive care unit.

a Total number of patients with a prescription using the Logipren^®^ CPOE in all NICUs during the study period (denominator used to calculate frequencies of patients with a prescription for each medication)

b At least 2 days, consecutive or not.

## Methods of Administration

**FIGURE 2:**
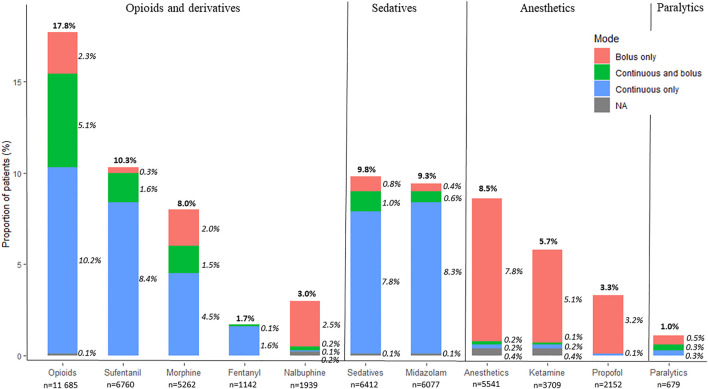
Proportion of neonates with a prescription for commonly used opioids, sedatives, anesthetics, and paralytics, and the methods of their administration (bolus only, continuous and bolus, or continuous only). NA corresponds to patients for whom the method of administration was not available. Percentages were calculated with the total number of patients with a prescription as the denominator (*n* = 65,555). Percentages may not sum up to 100% due to rounding.

Figure 2 depicts the methods of administration of the most commonly used substances. Among neonates who were prescribed opioids, 87% were prescribed a continuous infusion, including 29% with both continuous and bolus prescriptions. Among neonates who were prescribed midazolam, 96% were prescribed a continuous infusion, while hydroxyzine and diazepam were prescribed only for intermittent administrations. Among neonates who were prescribed anesthetics, 97% were prescribed single doses. Among neonates with a paralytic prescription, 46% were ordered as single doses, 28% as continuous infusion, and 26% as both continuous infusion and bolus.

### Associated Prescriptions

Among neonates prescribed an opioid, 50.4% had a concomitant prescription of a sedative, 54.6% of acetaminophen, and 5.3% of a paralytic. Most patients with a sedative prescription had a concomitant medication order: 91.8% for an opioid, 23.1% an anesthetic, and 8.4% a paralytic. Almost all neonates with a paralytic prescription had a concomitant prescription: 91.8% for opioids, 79.7% for sedatives, and 38.6% for anesthetics (Table 2).

**TABLE 2:** Number and proportion of patients with and/or without concomitant prescriptions by drug class.

Prescription Categories	Acetaminophen *n* = 24,395	Opioids *n* = 11,685	Sedatives *n* = 6,412	Anesthetics *n* = 5,541	Paralytics *n* = 679	Antidotes *n* = 473
Number of patients without concomitant prescription, *n* (%)	17,324 (71.0)	2002 (17.1)	200 (3.1)	1,626 (29.3)	9 (1.3)	91 (19.2)
Number of patients with and without concomitant prescription, *n* (%) *	5,868 (24.1)	4,363 (37.3)	454 (7.1)	860 (15.5)	9 (1.3)	40 (8.5)
Number of patients with concomitant prescription, *n* (%)	1,203 (4.9)	5,320 (45.5)	5,758 (89.8)	3,055 (55.1)	661 (97.3)	342 (72.3)
Number of patients with concomitant prescription by drug class, *n* (%)						
Opioids	6376 (26.1)	-	5,888 (91.8)	3,174 (57.3)	623 (91.8)	301 (63.6)
Sedatives	3,150 (12.9)	5,888 (50.4)	-	1,480 (26.7)	541 (79.7)	252 (53.3)
Anesthetics	2,183 (8.9)	3,174 (27.2)	1,480 (23.1)	-	262 (38.6)	76 (16.1)
Paralytics	284 (1.2)	623 (5.3)	541 (8.4)	262 (4.7)	-	14 (3.0)
Antidotes	263 (1.1)	301 (2.6)	252 (3.9)	76 (1.4)	14 (2.1)	-
acetaminophen	-	6,376 (54.6)	3,150 (49.1)	2,183 (39.4)	284 (41.8)	263 (55.6)

Percentages were calculated with the total number of patients per drug class.*Patients could have multiple prescriptions for one drug class: some with and some without concomitant prescriptions of another drug class

### Prescribed Doses

For most drugs prescribed as continuous infusions, physicians ordered a loading dose at the start of treatment for a minority of neonates. For opioids 12.8, 1.7, and 1.9% of neonates were prescribed a loading dose prior to a continuous infusion of sufentanil, morphine, and fentanyl, respectively. For sedatives 2.4, 0.7, and 65.9% of neonates were prescribed a loading dose prior to a continuous infusion of midazolam, clonidine, and dexmedetomidine, respectively. For anesthetics 5.5, 7.8, and 33.3% of neonates were prescribed a loading dose prior to a continuous infusion of ketamine, propofol, and thiopental, respectively. For atracurium 38.5% of neonates were prescribed a loading dose prior to a continuous infusion. We observed variations in initial, maintenance, and cumulative doses used and duration of treatment for each drug. Detailed prescription characteristics (loading doses, doses, and durations of treatment) are described in Table 3 for drugs used as continuous infusions and Table 4 for intermittent administration and single doses.

**TABLE 3:** Description of doses and duration of treatment for drugs prescribed as continuous infusion

Molecules, N (%) *n*=*n*. patients with continuous infusion/N patients with a prescription of the molecule	N patients with a loading dose (%)	Loading dose (dose/kg)	Initial dose (dose/kg)	Mean maintenance dose (dose/kg/h)	Cumulative dose (dose/kg)	Duration of treatment (days)
		Mean (SD)	Mean (SD)	Mean (SD)	Mean (SD)	Mean (SD)
		Median (IQR)	Median (IQR)	Median (IQR)	Median (IQR)	Median (IQR)
		p2.5 – p97.5	p2.5 – p97.5	p2.5 – p97.5	p2.5 – p97.5	p2.5 – p97.5
Opioids and derivatives						
Sufentanil (µg/kg) *n*=6580/6751 (97.5%)	842 (12.8%)	0.40 (0.73)	0.23 (0.33)	0.27 (0.23)	50.9 (131.6)	6.6 (8.9)
		0.2 (0.2-0.5)	0.2 (0.2-0.2)	0.2 (0.18 - 0.31)	16.0 (6.0–43.6)	4 (2-7)
		0.1–0.6	0.05–0.5	0.05 - 0.84	1.2–314.6	1–33
Morphine (µg/kg) *n*=3934/5213 (75.5%)	66 (1.7%)	32.5 (16.5)	18.5 (28.5)	24.6 (116.8)	5089 (32123)	7.0 (8.8)
		25 (20–50)	10 (10-20)	19.1 (10-27.3)	1427 (480-4137)	4 (2-8)
		15–54	5–50	5 - 64.9	113 - 26638	1–33
Fentanyl (µg/kg) *n*=1122/1141 (98.3%)	21 (1.9%)	1.0 (0.5)	0.80 (0.47)	1.1 (0.6)	236 (404)	8.2 (10.0)
		1 (1–1)	0.5 (0.5-1)	1 (0.5-1.4)	86 (25–255)	5 (2-9)
		0.5–2	0.2– 2	0.3 - 2.5	4.8–1316	1–34
Remifentanil (µg/kg) n=24/24 (100%)	17 (70.8%)	0.65 (0.23)	29.3 (15.7)	29.4 (15.0)	690.5 (618)	1.4 (0.6)
		0.5 (0.5–1)	30 (30-30)	30 (30-30)	720 (200-758)	1 (1-2)
		0.5–1	0.06 - 60	0.3–60	6.2–2033	1–2.4
Nalbuphine (mg/kg) *n*=159/1814 (8.8%)	4 (2.5%)	0.13 (0.09)	0.05 (0.09)	0.06 (0.09)	2.7 (3.6)	2.6 (1.8)
		0.15 (0.08–0.2)	0.05 (0.02-0.05)	0.05 (0.03-0.05)	1.6 (1.0 – 3.0)	2 (1-3)
		0.03–0.2	0.015 - 0.18	0.017–0.18	0.2–13	1–7
Sedatives						
Midazolam (µg/kg) *n*=5808/6052 (96.0%)	140 (2.4%)	76 (64)	46.9 (880)	40.3 (43.4)	8547 (32850)	7.1 (10.2)
		50 (50–100)	30 (20-30)	30 (20-47)	2234 (919 - 6484)	4 (2-8)
		10–200	6–100	6.3–136	144 – 53928	1 – 34
Clonidine (µg/kg) *n*=147/168 (87.5%)	1 (0.7%)	1	0.42 (0.18)	0.55 (0.27)	193.1 (307.6)	12.3 (12.3)
			0.5 (0.3-0.5)	0.5 (0.38-0.7)	78.5 (29.2 - 218.3)	8 (4-16.5)
			0.02–0.76	0.12–1.02	9.4 - 866	2 - 37
Dexmedetomidine (µg/kg) *n*=41/41 (100%)	27 (65.9%)	0.05 (0.01)	0.093 (0.09)	0.17 (0.08)	38.1 (38.6)	8.9 (6.6)
		0.05 (0.05-0.05)	0.05 (0.05-0.1)	0.17 (0.11-0.2)	24.0 (15.7-51.6)	6 (5-12)
		0.04 – 0.05	0.05–0.2	0.05–0.28	1.5–133	1 - 25
Anesthetics						
Ketamine (mg/kg) *N* = 165/3498 (4.7%)	9 (5.5%)	0.9 (0.67)	0.9 (2.2)	1.16 (3.6)	190.9 (498.7)	8.0 (13.2)
		0.5 (0.5-1)	0.5 (0.12-1)	0.57 (0.3-1.0)	33.2 (12-172.8)	4 (1-10)
		0.3–2	0.04 - 3.8	0.05 - 3.3	1.0 - 973	1 - 42
Propofol (mg/kg) *N* = 64/2131 (3.0%)	5 (7.8%)	1.6 (0.9)	2.8 (2.8)	3.0 (2.8)	200 (682)	3.5 (9.7)
		1 (1-2)	2 (1-3)	2 (1-3.1)	48 (24-164)	1 (1-2)
		1–2.9	0.5 - 9	0.6 - 9	1.8 - 1101	1 - 22
Thiopental (mg/kg) *n*=18/47 (38.3%)	6 (33.3%)	3.5 (0.837)	5.9 (18.5)	6.0 (18.5)	317 (887)	2.6 (1.9)
		3 (3-3.75)	1 (1-2)	1.6 (1-2.2)	48 (24.8 -236)	2 (1-2.8)
		3–4.9	0.1 - 48	0.1 - 48	0.6 - 2403	1 - 6.6
Paralytics						
Atracurium (mg/kg) *n*=353/585 (60.3%)	136 (38.5%)	0.29 (0.11)	0.37 (0.12)	0.43 (0.2)	38.2 (106.5)	3.3 (3.3)
		0.25 (0.250.3)	0.4 (0.3-0.4)	0.4 (0.3–0.47)	15.9 (9.6-32.3)	2 (2-4)
		0.22–0.5	0.2 - 0.6	0.2–1	1.75–215.7	1–10.2
Rocuronium (mg/kg) *n*=14/23 (60.9%)	3 (21.4%)	0.25 (0.3)	0.3	0.35 (0.10)	26.9 (30.8)	3.4 (2.3)
		0.075 (0.075-0.34)		0.3 (0.3–0.365)	17.4 (13.2–25.2)	3 (2–3.7)
		0.075–0.57		0.3–0.6	4.0 – 101	1–8.3
Vecuronium (mg/kg) *n*=6/8 (75%)	4 (66.6%)	0.01	0.05	0.61 (0.66)	0.05	1.2 (0.41)
				0.61 (0.01–1.2)		1 (1–1)
				0.006–1.2		1–1.9
Antidotes						
Naloxone (µg/kg) *n*=12/452 (2.7%)	4 (33.3%)	32.5 (45)	6.8 (14.1)	6.7 (14.1)	293.1 (645.0)	2.25 (1.8)
		10 (10–32.5)	2 (0.24–6.25)	2 (0.24–5.0)	49.5 (8.5–220.8)	1.5 (1–2.5)
		10–93	0.06–39	0.06–39	0.3–1783	1–5.7
Flumazenil (µg/kg) *n*=9/28 (32.1%)	9 (100%)	0.01	0.005	0.005	0.09 (0.05)	1.2 (0.4)
					0.1 (0.05–0.12)	1 (1–1)
					0.007–0.16	1–2

Results are presented as mean (standard deviation), median (interquartile) and 2.5 – 97.5 percentile range (to exclude extreme values).

**TABLE 4:** Description of doses and duration of treatment for drugs prescribed as single doses or intermittent administration

Molecule n=n. patients with intermittent administration/N patients with a prescription of the molecule (%)	Route	Unit dose (dose/kg)	Dose interval (h) (% of patients)	Mean daily dose (dose/kg/d)^*^	Cumulated dose (dose/kg)^*^	Duration of treatment (days)^*^
		Mean (SD)		Mean (SD)	Mean (SD)	Mean (SD)
		Median (IQR)		Median (IQR)	Median (IQR)	Median (IQR)
		p.2.5-p.97.5		p.2.5-p.97.5	p.2.5-p.97.5	p.2.5-p.97.5
Acetaminophen (mg/kg) *n*= 17327/24395 (71.0%)	PO	13.4 (2.4)	6h (83%), 8h (21%), 12h (3%), other < 1%	51 (11.3)	379 (590)	8.3 (12.3)
		15 (10-15)		60 (40–60)	181 (93-414)	4 (2-9)
		10–15		30–60	32–1971	1–44
Acetaminophen (mg/kg) *n*=15181/24395 (62.2%)	IV	11.7 (4.4)	6h (63%), 12h (30%), 8h (15%), other < 1%, Single dose (14%)	31.9 (10.2)	200 (333)	7.0 (9.9)
		10 (10-15)		30 (25.5–0)	107 (54–223)	4 (2-8)
		7.5–20		20–60	20-943	1–33
Opioids						
Morphine (µg/kg) *n*=2034/5213 (39.0%)	PO	132 (202)	6 h (61%), 4 h (55%), 8 h (15%), 12 h (11%), 3 h (8%), other <5%	630 (867)	5330 (12030)	8.6 (11.4)
		88 (50-167)		480 (312-800)	2066 (896 – 4842)	5 (2-11)
		12.5 - 500		80 - 1947	97-32343	1 - 34
Sufentanil (µg/kg) *n*=401/6751 (5.9%)	IV	0.3 (0.61)	Single dose (100%)			
		0.2 (0.2-0.2)				
		0.1 – 0.5				
Morphine (µg/kg) *n*=285/5213 (5.5%)	IV	41.9 (37.5)	8h (41%), 4h (27%), 6h (17%), 24h (8%), other <5%, Single dose (11.2%)	276 (595)	641.2 (2683)	2.6 (6.9)
		50 (20-50)		150 (90-250)	150 (63-360)	1 (1-2)
		5 – 120.8		11.5 - 1200	1.1-3116	1 - 8.7
Fentanyl (µg/kg) *n*=29/1141 (2.5%)	IV	0.9 (0.5)	Single dose (100%)			
		1 (0.5-1)				
		0.3 - 2				
Nalbuphine (mg/kg) *n*=1561/1814 (86.0%)	IV	0.21 (1.25)	6h (62%), 4h (5%), other <5%, Single dose (35.2%)	0.5 (0.3)	1.2 (1.5)	2.9 (2.3)
		0.2 (0.1-0.2)		0.4 (0.2-0.8)	0.8 (0.35-1.4)	2 (1-3)
		0.03 - 0.6		0.15 - 1.1	0.1-5.8	1 - 9
Nalbuphine (mg/kg) *n*=300/1814 (16.5%)	IR	0.2 (0.05)	6h (90%), 24h (7%), 4h (2%), 8h (<1%), Single dose (3.3 %)	0.74 (0.18)	1.7 (2.4)	2.6 (4.2)
		0.2 (0.2-0.2)		0.8 (0.8-0.8)	1 (0.8-2.1)	2 (1-3)
		0.1 - 0.4		0.2 - 0.8	0.2-5.8	1 – 8.8
Sedatives						
Midazolam (µg/kg) *n*=331/6052 (5.5%)	IV	65.7 (44.5)	3h (12%), other < 1%, Single dose (86.4 %)	510.8 (402.3)	1095 (1670)	2.6 (2.1)
		50 (50-100)		400 (160-800)	720 (400-1062)	2 (1-3)
		10 - 200		52.5 - 1416	29 - 4794	1 - 9
Midazolam (µg/kg) *n*=25/6052 (0.4%)	PO	232 (154)	12 h (68%), 6 h (52%), 3h (8%), 8h (8%), 1 h (< 1%)	764 (448)	2727 (2859)	4.1 (3.5)
		200 (105 -300)		687 (459-1200)	1200 (613-4717)	3 (1-6)
		(25 - 578)		120 - 1511	51-8399	1 - 11
Midazolam (µg/kg) *n*=136/6052 (2.2%)	IR	172 (128)	6 h (1%), 12 h (2%), 3h (<1%), 24h (1%), Single dose (97.1 %)	233 (121)	252 (263)	1.25 (0.5)
		150 (100-300)		220 (175-278)	161 (76-337)	1 (1-1.25)
		10 - 395		(107 - 380)	61-599	1 - 1.9
Midazolam (µg/kg) *n*=15/6052 (0.2%)	IN	129 (130)	6 h (60%), 24 h (40%)	263.3 (161)	670 (1646)	2 (2.8)
		100 (90-100)		220 (100-400)	304 (100-400)	1 (1-1.5)
		41.5 - 500		100 - 511	38-4443	1 - 8.9
Hydroxyzine (mg/kg) *n*=355/359 (98.9%)	PO	1.1 (0.9)	12 h (39%), 24 h (26%), 8 h (19%), 6 h (3%), other <1%, Single dose (23.4 %)	1.2 (0.6)	11.6 (27)	9.7 (15.4)
		1 (0.5-1)		1 (1-1.3)	4 (1.7-11.1)	4 (2-10.3)
		0.25 - 4		0.5 - 3	0.4-70.5	1 - 61.4
Hydroxyzine (mg/kg) *n*=8/359 (2.2%)	IV	1.5 (0.6)	12h (63%), 8h (12%), Single dose (25 %)	1.3 (0.8)	4.3 (2.8)	3.8 (1.2)
		2 (1-2)		1 (1-1)	3.5 (2.5-4.7)	4 (3.3-4.7)
		0.5 - 2		1 - 2.75	1.9-8.9	2.1 - 5
Diazepam (mg/kg) *n*=20/332 (6.0%)	IV	0.5	Single dose (100%)			
Diazepam (mg/kg) *n*=308/332 (92.8%)	PO	0.35 (0.53)	6h (71%), 8h (20%), 4h (13%), 12h (10%), other <5%	1.0 (0.96)	7.7 (16.5)	7.5 (11)
		0.25 (0.16-0.33)		1 (0.66-1)	2.6 (1.1-6.8)	3 (2-8)
		0.08 - 1.8		0.27 - 2.6	0.4-56	1 - 40
Diazepam (mg/kg) *n*=43/332 (13.0%)	IR	0.5 (0.03)	Single dose (100%)			
		0.5 (0.5-0.5)				
		0.5 - 0.5				
Clonidine (µg/kg) *n*=43/168 (25.6%)	PO	2.9 (2.3)	6h (100%), 4h (5%), 12h (5%)	9.25 (4.2)	148 (317)	13.4 (13)
		2 (1.5-3.3)		8 (6.9-9.5)	69 (47-140)	9 (6-15)
		0.5 - 9.0		5.4 - 21.5	24-336	3 - 35
Anesthetics						
Ketamine (mg/kg) *n*=3294/3498 (94.2%)	IV	1.1 (0.6)	Single dose (100%)			
		1 (0.5-2)				
		0.5 - 2				
Ketamine (mg/kg) *n*=109/3498 (3.1%)	IR	1.1 (1.1)	Single dose (100%)			
		0.5 (0.5-1)				
		0.5 – 5				
Ketamine (mg/kg) *n*=11/3498 (0.3%)	IN	1.6 (0.5)	Single dose (100%)			
		2 (1-2)				
		1 – 2				
Ketamine (mg/kg) *n*=5/3498 (0.1%)	IM	5.6 (2.4)	Single dose (100%)			
		5 (5-8)				
		2 – 8				
Propofol (mg/kg) *n*=2079/2131 (97.6%)	IV	1.1 (0.5)	Single dose (100%)			
		1 (1-1)				
		0.5 - 3				
Thiopental (mg/kg) *n*=33/47 (70.2%)	IV	2.6 (0.6)	Single dose (100%)			
		2.5 (2.5-3)				
		0.5 - 4				
Paralytics						
Atracurium (mg/kg) *n*=270/585 (46.2%)	IV	0.31 (0.1)	Single dose (100%)			
		0.3 (0.25-0.3)				
		0.25 - 0.5				
Suxamethonium (mg/kg) *n*=103/103 (100%)	IV	1.5 (0.4)	Single dose (100%)			
		1.5 (1.5-1.5)				
		0.6 – 2				
Rocuronium (mg/kg) *n*=9/23 (39.1%)	IV	0.8 (0.25)	Single dose (100%)			
		0.85 (0.6-1)				
		0.3 - 1				
Vecuronium (mg/kg) *n*=2/8 (25%)	IV	0.01	Single dose (100%)			
Antidotes						
Naloxone (µg/kg) *n*=432/452 (95.6%)	IV	24.9 (35.3)	Single dose (100%)			
		10 (10-10)				
		1 - 100				
Naloxone (µg/kg) *n*=11/452 (2.5%)	SC	34.2 (40.7)	Single dose (100%)			
		10 (10-77.5)				
		10 - 100				
Naloxone (µg/kg) *n*=7/452 (1.5%)	IM	12.1 (7.2)	Single dose (100%)			
		10 (10-10)				
		10 - 28				
Flumazenil (µg/kg) *n*=19/28 (67.9%)	IV	0.014 (0.01)	Single dose (100%)			
		0.01 (0.01-0.01)				
		0.01 - 0.03				

* Mean daily dose, cumulative dose and duration of treatment are described only for intermittent administration.

Doses and duration of treatment are presented as mean (standard deviation), median (interquartile) and 2.5 – 97.5 percentile range (to exclude extreme values).

IV, intravenous; PO, per os; IM, intramuscular; SC, subcutaneous; IN, intranasal.

The total number of patients indicated for each molecule and used to calculate proportions is the total number of patients for whom we had data on dosing regimen.

87 neonates received acetaminophen by rectal route (0.4%) without detailed data on the dose prescribed.

### Comparisons Between Gestational Age Groups

**FIGURE 3:**
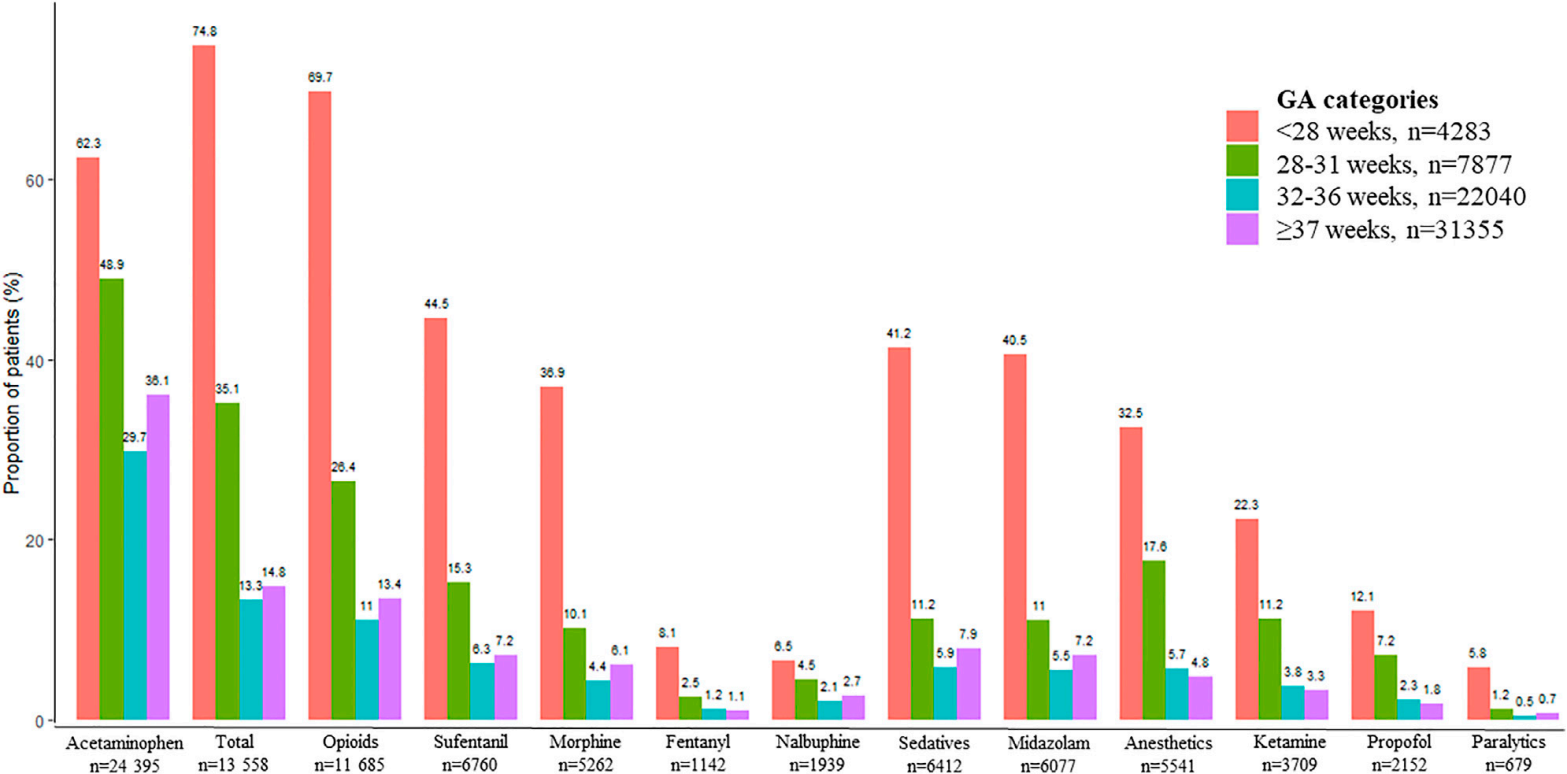
Proportion of patients for each drug class or molecule according to gestational age (GA) group. Percentages were calculated with the total number of patients in each GA group as the denominator. “Total” represents the number of patients with at least one prescription of an analgesic, sedative, paralytic, and/or anesthetic agent except acetaminophen.

The frequencies of prescriptions of an analgesic, sedative, anesthetic, and/or paralytic were 3,771/4,283 (88.0%), 4,955/7,877 (62.9%), 7,681/22,040 (34.9%) and 12,933/31,355 (41.2%) for infants born before 28 weeks, between 28 and 31 weeks, between 32 and 36 weeks, and at ≥ 37 weeks, respectively. After excluding acetaminophen these frequencies were 3,204/4,283 (74.8%), 2,764/7,877 (35.1%), 2,934/22,040 (13.3%), and 4,656/31,355 (14.8%) in infants born before 28 weeks, between 28 and 31 weeks, between 32 and 36 weeks, and at ≥ 37 weeks, respectively (Figure 3).

For opioids and sedatives commonly administered by continuous infusion (sufentanil, morphine, fentanyl, and midazolam), infants born before 28 weeks were prescribed significantly lower initial doses, significantly higher cumulative doses, and were treated for significantly longer periods than all other GA groups (*p*-values < 0.001). Maintenance doses of sufentanil, fentanyl, and midazolam were higher for term neonates (≥37 weeks) than other GA groups (*p*-values < 0.001). Prescriptions for atracurium did not differ significantly by GA, except for a lower initial dose for neonates born before 28 weeks (*p*-value < 0.001) (Table 5).

**TABLE 5:** Comparison of initial dose, cumulative dose, maintenance dose, and duration of treatment by gestational age groups for drugs commonly prescribed as continuous infusions

Gestational age (weeks)	< 28	28-31	32-36	≥ 37	Total	P*	Description of significant differences between GA groups†
Sufentanil	*n*=1824	*n*=1160	*n*=1365	*n*=2231	*n*=6580		
Initial dose (µg/kg/h)	0.2 (0.1-0.2)	0.2 (0.1-0.2)	0.2 (0.2-0.2)	0.2 (0.2-0.2)	0.2 (0.2-0.2)	<.001	The higher the GA, the higher the initial dose (*P*-values<.01), except for <28 wks and 28-31 wks (*P*-value > .99)
Cumulative dose (µg/kg)	25.8 (9.3-75.3)	10.0 (4.8-29.0)	12.2 (4.9-30.4)	16.6 (6.8-37.8)	16.0 (6.0 - 43.6)	<.001	Cumulative dose for < 28 wks higher than all other groups (*P*-values<.001). Cumulative dose for ≥ 37wks higher than 28-31 and 32-36 wks (*P*-values<.001).
Maintenance dose (µg/kg/h)	0.2 (0.17-0.31)	0.2 (0.15-0.25)	0.2 (0.18-0.30)	0.22 (0.2-0.36)	0.2 (0.18 - 0.31)	<.001	Maintenance dose for ≥ 37 wks higher than others (*P*-values<.001). Maintenance dose for 28-31 wks lower than others (*P*-values<.001).
Duration of treatment (d)	6.0 (3-12)	3 (2-6)	3 (2-5)	3 (2-6)	4 (2-7)	<.001	Duration of treatment for < 28 wks longer than others (*P*-values<.001). Duration of treatment for ≥ 37 wks longer than 28-31 and 32-36 wks (*P*-values=.03).
Morphine	*n*=1311	*n*=673	*n*=700	*n*=1250	*n*=3934		
Initial dose (µg/kg/h)	10 (10-20)	10 (10-20)	10 (10-20)	20 (10-20)	10 (10-20)	<.001	Initial dose for ≥ 37 wks higher than others (*P*<.001 vs <28 wks, *P*=.02 vs 28-31 wks and *P*=.003 vs 32-36 wks). Initial dose for < 28 wks lower than others (*P*=.004 vs 28-31 wks, *P*=.02 vs 32-36 wks, *P*<.001 vs >36 wks).
Cumulative dose (µg/kg)	2601 (848-6750)	1207 (438-3678)	1004 (432-2819)	934 (434-2679)	1427 (480-4137)	<.001	Cumulative dose for < 28 wks higher than others (*P*-values<.001).
Maintenance dose (µg/kg/h)	19.4 (10-28.8)	18.6 (10-25)	16.8 (10-25)	20 (10-29)	19.1 (10-27.3)	.006	Maintenance dose for < 28 wks higher than 32-36 wks (*P*=.02).
Duration of treatment (d)	7 (3-15)	4 (2-8)	3 (2-6)	3 (2-5)	4 (2-8)	<.001	The lower the GA, the longer the duration of treatment (*P*-values<.001 for all, *P*=0.03 between 28-31 and 32-36 wks), except for 32-36 and ≥ 37 wks (*P*=.13).
Fentanyl	*n*=344	*n*=196	*n*=263	*n*=319	*n*=1122		
Initial dose (µg/kg/h)	0.5 (0.5-0.62)	0.5 (0.5-1)	0.75 (0.5-1)	1 (0.5-1)	0.5 (0.5-1)	<.001	Initial dose for ≥ 37 wks higher than others (*P*-values<.001). Initial dose for < 28wks lower than others (*P*-values<0.001).
Cumulative dose (µg/kg)	163 (47-573)	72 (28-240)	55 (24-154)	74 (24-210)	86 (25-255)	<.001	Cumulative dose of < 28wks higher than others (*P*-values<0.001). Cumulative dose of 28-32wks higher than 32-36wks (*P*=0.04).
Maintenance dose (µg/kg/h)	0.9 (0.5-1.4)	0.9 (0.5-1.4)	0.9 (0.5-1.1)	1 (0.74-1.6)	1 (0.5-1.4)	<.001	Maintenance dose of ≥ 37 wks higher than others (*P*-values<0.01).
Duration of treatment (d)	8 (3-19)	4 (2-10)	3 (2-6)	3 (2-7)	5 (2-9)	<.001	Duration of treatment of < 28wks longer than others (*P*-values<0.001). Duration of 28-31 wks longer than 32-36 (*P*=.01) and ≥ 37 wks (*P*<.001).
Midazolam	*n*=1693	*n*=818	*n*=1154	*n*=2143	*n*=5808		
Initial dose (µg/kg/h)	20 (20-30)	30 (20-30)	30 (20-30)	30 (20-50)	30 (20-30)	<.001	The lower the GA, the lower the initial dose (*P*-values<.001 for all, except <28 and 28-32 wks vs *P*=.04).
Cumulative dose (µg/kg)	3236 (1200-9821)	1688 (730-5074)	1773 (789-4837)	2161 (953-5584)	2234 (919-6484)	<.001	Cumulative dose for < 28 wks higher than others (*P*-values<.001). Cumulative dose for ≥ 37 wks higher than 28-32 wks (*P*=0.01) and 32-36 wks (*P*=.005).
Maintenance dose (µg/kg/h)	30 (20-40)	30 (20-38)	30 (20-45)	31 (22-59)	30 (20-47)	<.001	Maintenance dose for ≥ 37 wks higher than others (*P*-values<.001). Maintenance dose of 32-36 wks higher than < 28 (*P*=.04) and 28-31 wks (*P*=.003).
Duration of treatment (d)	6 (3-13)	3 (2-7)	3 (2-6)	3 (2-6)	4 (2-8)	<.001	Duration of treatment for < 28 wks longer than others (*P*-values<.001).
Atracurium	*n*=107	*n*=39	*n*=61	*n*=146	*n*=353		
Initial dose (mg/kg/h)	0.3 (0.3-0.4)	0.4 (0.3-0.4)	0.4 (0.3-0.4)	0.4 (0.3-0.4)	0.4 (0.3-0.4)	<.001	Initial dose for < 28 wks lower than 32-36 wks and ≥ 37 wks (*P*-values<.001). No difference between other groups.
Cumulative dose (mg/kg)	19.7 (9.6-34)	15.9 (9.6-27.4)	17.9 (9.6-43)	14.1 (9.6 -30.1)	15.9 (9.6-32.3)	.56	
Maintenance dose (mg/kg/h)	0.4 (0.3-0.44)	0.4 (0.3 -0.46)	0.4 (0.36-0.5)	0.4 (0.34-0.48)	0.4 (0.3-0.47)	.03	No difference between groups.
Duration of treatment (d)	3 (2-4)	3 (2-4)	2 (1-4)	2 (2-3)	2 (2-4)	.30	

GA: gestational age in weeks (wks), d: days. Doses and duration of treatment are described by their medians (interquartile range).

*p-value from Kruskal-Wallis test. †Description of post-hoc analysis with Wilcoxon signed-rank test and Bonferroni correction.

Oral acetaminophen prescriptions were at higher unit doses and higher mean daily doses in term neonates, while the lower the GA, the longer the prescription duration and the higher the cumulative dose (*p*-values < 0.001). Intravenous acetaminophen, on the other hand, was prescribed at a lower unit dose but higher mean daily dose (explained by the shorter dose intervals) in term neonates (*p*-values < 0.001) (Table 6). Oral morphine prescriptions did not differ significantly between GA groups in unit dose (*p* = 0.06), but the mean daily dose was higher for term neonates than for neonates born before 32 weeks (*p* = 0.002), and prescription duration was significantly higher for those born before 32 weeks as compared to those born at or after 32 weeks (*p* < 0.001). Term neonates were prescribed significantly higher unit doses of anesthetics (propofol and ketamine) and sufentanil when used as a single dose than preterm neonates (*p*-values < 0.001) (Table 6).

**TABLE 6:** Comparison of doses (unit doses, mean daily doses, and cumulative doses) and duration of treatment by gestational age for drugs commonly prescribed as a bolus (intermittent administration or single dose).

Gestational age (weeks)	< 28	28-31	32-36	≥ 37	Total	p*	Description of significant differences between GA groups†
Intermittent administration							
Oral acetaminophen	*n*=1643	*n*=2451	*n*=4557	*n*=8673	*n*=17327		
Unit dose (mg/kg)	15 (10-15)	10 (10-15)	10 (10-15)	15 (15-15)	15 (10-15)	< 001	Unit dose for ≥ 37wks higher than other groups (*P*-values<.001). Unit dose of < 28 wks higher than 28-32 and 32-36 wks (*P*-values<.001).
Mean daily dose (mg/kg/d)	40 (30-60)	37 (30-60)	40 (40-60)	60 (60-60)	60 (40-60)	< 001	Daily dose for ≥ 37wks higher than others (*P*-values<0.001). 32-36 wks higher than < 28 and 28-31 wks, < 28 wks higher than 28-31 wks (*P*-values<.001).
Cumulative dose (mg/kg)	359 (121-988)	217 (90-632)	191 (89-466)	165 (91-301)	181 (93-414)	< 001	Differences between all groups: the lower the GA, the higher the cumulative dose (*P*-values<.001).
Duration of treatment (d)	9 (3-24)	6 (3-18)	5 (2-11)	3 (2-6)	4 (2-9)	< 001	Differences between all groups: the lower the GA, the higher the longer of treatment (*P*-values<.001).
IV acetaminophen	*n*=2069	*n*=2436	*n*=3221	*n*=5286	*n*=13012		
Unit dose (mg/kg)	10 (10-15)	10 (10-15)	10 (10-20)	7.5 (7.5-10)	10 (10-15)	<.001	Unit dose for ≥ 37wks lower than others (*P*-values<.001).
Mean daily dose (mg/kg/d)	23 (20-35)	20 (20-29)	30 (30-40)	30 (30-40)	30 (25.5-40)	<.001	Daily dose for ≥ 37wks higher than others, 32-36 wks higher than < 28 wks and 28-31 wks, < 28 wks higher than 28-31 wks (*P*-values<.001).
Cumulative dose (mg/kg)	212 (90-409)	122 (60-240)	110.5 (58-209)	80 (44-157)	107 (54-223)	<.001	Differences between all groups: the lower the GA, the higher the cumulative dose (*P*-values<.001, except between 28-31 wks and 32-36 wks *P*=.02).
Duration of treatment (d)	7 (4-16)	6 (3-10)	4 (2-7)	3 (2-5)	4 (2-8)	<.001	Differences between all groups: the lower the GA, the longer the duration of treatment (*P*-values<.001).
Oral morphine	*n*=619	*n*=201	*n*=344	*n*=870	*n*=2034		
Unit dose (µg/kg)	100 (50-167)	83 (50-150)	83 (50-167)	83 (50-167)	88 (50-167)	.06	
Mean daily dose (µg/kg/d)	477 (277-729)	417 (295-628)	480 (340-778)	480 (320-800)	480 (312-800)	.002	Daily dose for ≥ 37wks higher than < 28 wks (*P*=.02) and 28-31 wks (*P*=.01).
Cumulative dose (µg/kg)	2417 (949-5763)	2198 (975-4848)	1820 (840-3906)	1945 (863-4781)	2066 (896-4842)	.049	Cumulative dose for < 28 wks higher than 32-36 wks (*P*=.04).
Duration of treatment (d)	7 (3-12.5)	6 (3-12)	4 (2-9)	5 (2-10)	5 (2-11)	<.001	Duration of treatment for < 28 wks higher than 32-36 wks and ≥ 37wks (*P*-values<.001). Duration of treatment for 28-31 wks higher than 32-36 wks (*P*=.009) and ≥ 37 wks (*P*=.04).
IV morphine	*n*=74	*n*=51	*n*=62	*n*=66	*n*=253		
Unit dose (µg/kg)	39 (10-50)	50 (25-50)	50 (20-50)	50 (20-50)	50 (20-50)	.04	No difference between groups.
Mean daily dose (µg/kg/d)	120 (40-196)	150 (110-300)	150 (127-300)	150 (150-240)	150 (90-250)	.002	Mean daily dose for < 28 wks lower than others *P*=.04 vs 28-31 wks, *P*=.01 vs 32-36 wks and *P*=.007 vs ≥ 37 wks).
Cumulative dose (µg/kg)	150 (51-312)	169 (86-380)	178 (69-390)	150 (93-358)	150 (63-360)	.46	
Duration of treatment (d)	1 (1-3)	1 (1-2)	1 (1-2)	1 (1-2)	1 (1-2)	.50	
Single doses							
Ketamine	*n*=846	*n*=817	*n*=753	*n*=878	*n*=3294		
Unit dose (mg/kg)	1 (0.5-2)	1 (0.5 -1)	1 (0.5-2)	1 (0.5-2)	1 (0.5-2)	<.001	Unit dose for ≥ 37wks higher than other groups (*P*-values<.01), 28-31 wks lower than < 28 wks (*P*=.01) and 32-36 wks (*P*<.001).
Propofol	500	551	496	532	n=2079		
Unit dose (mg/kg)	1 (1-1)	1 (1-1)	1 (1-1)	1 (1-1)	1 (1-1)	<.001	Unit dose for ≥ 37wks higher than others (*P*=.01 vs 32-36 wks and *P*-values<.001 vs others). Unit dose of 32-36 wks higher than 28-31 wks (*P*=.001).
Sufentanil	*n*=202	*n*=84	*n*=59	*n*=56	*n*=401		
Unit dose (µg/kg)	0.2 (0.2-0.2)	0.2 (0.2-0.2)	0.2 (0.2-0.2)	0.2 (0.2-0.5)	0.2 (0.2-0.5)	<.001	Unit dose for ≥ 37wks higher than <28 wks (*P*<0.001), 28-31 wks (*P*=0.03) and 32-36 wks (*P*=.007).
Atracurium	*n*=136	*n*=44	*n*=36	*n*=54	*n*=270		
Unit dose (mg/kg)	0.3 (0.3-0.3)	0.3 (0.25-0.3)	0.3 (0.25-0.32)	0.3 (0.25-0.3)	0.3 (0.25-0.3)	.73	

GA: gestational age in weeks (wks), d: days, IV: intravenous. Doses and duration of treatment are described by their medians (interquartile range).

*P-value by the Kruskal-Wallis test. †Description of post-hoc analysis with Wilcoxon signed-rank test and Bonferroni correction.

## Discussion

In this study, we reported frequencies of prescription, methods of administration, concomitant drug prescriptions, and dosing of analgesics, sedatives, anesthetics, and paralytics in a large cohort of neonates and according to their GA. In our cohort, 44.8% of all neonates and 88.0% of preterm neonates born before 28 weeks were prescribed at least one of the drugs we studied during their NICU stay. Because our cohort represented almost half of French NICUs (30 NICUs out of 67, 45%) over a 6-year period, it offers a pharmacoepidemiologic overview of sedation and analgesia prescription practices in neonates requiring intensive care.

In our study, 17.8% of patients were prescribed opioids, 9.8% sedatives, 8.5% anesthetics, and 1% paralytics. A prospective European study of preterm and term neonates reported that 26% received opioids, 12% sedatives, 3% anesthetics, and 8% paralytics (Carbajal et al., 2015). In our cohort, 42% of preterm infants born before 32 weeks of GA had an opioid prescription, 22% a sedative, 23% an anesthetic, and 3% a paralytic. Previous studies of preterm infants found that opioids were prescribed to 22–41% of infants, sedatives to 13–24%, anesthetics to 4% of infants, and a paralytic to 13% (Mehler et al., 2013; Carbajal et al., 2015; Borenstein-Levin et al., 2017; Zimmerman et al., 2017).

Routine use of opioids or sedatives during mechanical ventilation is not currently recommended due to conflicting results on their usefulness and concerns about their short- and long-term adverse effects (American Academy of Pediatrics, 2000; American Academy of Pediatrics, 2006; Committee on fetus and newborn, 2016; Ng et al., 2017; Ancora et al., 2019; Bellù et al., 2021). Nonetheless, in most cohort studies, including this one, continuous infusion of opioids and sedatives remains a current practice (Mehler et al., 2013; Carbajal et al., 2015; Borenstein-Levin et al., 2017; Zimmerman et al., 2017).

A strength of our study is its detailed description of the doses used, unlike previous multicenter cohorts reporting sedation and analgesia (Carbajal et al., 2015; Borenstein-Levin et al., 2017; Zimmerman et al., 2017). Clinical studies do not currently provide definitive conclusion about neurodevelopmental outcomes and analgesic/sedative use (Rozé et al., 2008; Loepke, 2010; Lammers et al., 2014; McPherson and Grunau, 2014; Valkenburg et al., 2015; Ancora et al., 2017; de Tristan et al., 2021), but it seems likely that the doses used influence this outcome, as suggested by the possible negative association between treatment duration and neurodevelopmental outcomes (Puia-Dumitrescu et al., 2021). The follow-up study of a randomized controlled trial found that morphine use did not appear to affect cognition and behavior at 8 or 9 years and might even have had a positive effect on executive functions (de Graaf et al., 2013). The median cumulative morphine dose in that trial was 751 μg/kg (IQR 485-1185), while the patients in our study were prescribed a median cumulative dose of 1427 μg/kg (IQR 480-4137). In a cohort of preterm neonates who had received a median morphine dose of 790 μg/kg (IQR 120-950) in the neonatal period, no deleterious effect was observed on neurobehavior at 7 years of age (Steinhorn et al., 2015). Inversely, two studies have reported that higher morphine exposure in neonates was associated with impaired cerebellar growth and poorer neurodevelopment in early childhood and at school age (Ranger et al., 2014, 2015; Zwicker et al., 2016). They used doses higher than in our cohort (median cumulative dose of 1,910 μg/kg in one and mean cumulative dose of 2,300 μg/kg in the other). Exposure to painful procedures was also associated with poorer outcomes in these studies. Hence, judicious use of morphine requires balancing the negative effects of neonatal pain with the potential impact of high cumulative doses of morphine on outcomes. Similar observations have been reported for midazolam. In a cohort of preterm neonates, higher midazolam exposure predicted impaired hippocampal growth and lower cognitive scores at 18 months (Duerden et al., 2016). The median cumulative dose reported in that study was 6,610 μg/kg (IQR 4100–12700), compared with 2,234 μg/kg (IQR 919-6484) in our study. For drugs for which neurodevelopmental outcomes are a concern, knowledge of the cumulative doses prescribed is necessary to draw conclusions.

Another originality of our study is that it describes the intermittent prescription of some of the drugs. We can assume that drugs prescribed as single intravenous doses in our study were used to handle procedural pain, discomfort and/or stress. They were mainly ketamine (*n* = 3294) and propofol (*n* = 2079) for anesthetics, sufentanil (*n* = 401) for opioids, midazolam (*n* = 331) for sedatives, and atracurium (*n* = 270) for paralytics. Sufentanil was the most frequently prescribed opioid in French NICUs, and fentanyl’s use as a single dose was anecdotal (*n* = 29). Although published experience with sufentanil is less abundant than that with fentanyl in neonates, its use seems reasonable in premature infants, especially for endotracheal intubation (Durrmeyer et al., 2014, 2018; Tauzin et al., 2021) and has been preferred in France over fentanyl after a neonatal animal study suggested its benefits regarding white-matter damage (Laudenbach et al., 2001). Anesthetics appear to be preferred to other drug classes for procedural sedation in French NICUs. Doses prescribed for our patients corresponded to those recommended in the literature for ketamine (0.5–2 mg/kg) and propofol (1–2.5 mg/kg), with doses higher for term neonates (Anand, 2001; Ancora et al., 2019). Dose adjustment by GA is not reported in the literature for ketamine, and, to our knowledge, no pharmacokinetic-pharmacodynamic studies in neonates have ever sought to ascertain the minimal effective dose. The pharmacodynamics of propofol have been studied in neonates, and low doses around 1 mg/kg are favored (Smits et al., 2016), consistent with the doses prescribed for most of our patients.

In the literature and in our study, sedatives and opioids were prescribed more frequently for low-GA neonates than term infants (Mehler et al., 2013; Carbajal et al., 2015). Despite the absence of GA-appropriate dosing recommendations for most of these drugs, dosing adjustments by GA were observed in our cohort for morphine, fentanyl, and midazolam: usually lower doses for lower GA. Infants born at a lower GA, and especially extremely preterm infants, were nonetheless potentially more highly exposed, with higher cumulative doses and longer durations of prescription. Maturation and physiological development according to GA lead to major interindividual variability in the neonatal population. Thus, it is difficult to think that neonates with different GA, postnatal age or weight will need the same dosing regimen (Allegaert et al., 2014). Preterm infants also are at higher risk for neurodevelopmental impairment (Pierrat et al., 2021), and determination of the minimal effective doses according to GA through pharmacokinetic/pharmacodynamic and dose-finding studies is essential to optimize neurodevelopment (Smits et al., 2017).

One option for preserving neurodevelopment might be the use of potentially neuroprotective substances, such as alpha-2-agonists (Alam et al., 2017). In our study, 216 patients were prescribed this pharmaceutical class, although few or none were reported in earlier multicenter cohorts. These sedatives are frequently used in adult and pediatric ICUs (Grant et al., 2016) and could offer advantages for neonates: reduction of opioid and benzodiazepine use (Morton et al., 2021), neuroprotective effects in animal models, reduction of the duration of mechanical ventilation, and shorter time until full enteral feeding. Few studies have been performed so far in neonates, and further research on dosing and tolerance could help evaluate the interest and the place of these drugs in neonatal sedation and analgesia strategies (Chrysostomou et al., 2014; Romantsik et al., 2017; Dersch-Mills et al., 2019).

To develop individualized dosing regimen for these drugs and to rationalize their use, development programs should be conducted, including: knowledge of developmental pharmacology, maturational pharmacokinetics and pharmacodynamics; dose-finding studies to provide individualized doses; and evaluation and confirmation of these doses in prospective studies including long-term safety follow-up. The use of modelling tools such as physiologically based pharmacokinetic models and population pharmacokinetic/pharmacodynamic studies seems relevant in this process, probably combined with evaluation of the impact of pharmacogenetics (Smits et al., 2017; Hahn et al., 2019). The pharmacodynamic aspects of these evaluation seem essential for dose-finding studies but have to be relevant clinically and raise the problem of available and reliable monitoring tools for pain and sedation.

This study has several limitations. First, we had limited clinical data about these patients; for example, we did not know their ventilatory status, the exact indication for treatment or the total duration of NICU stay. Thus, we could not estimate the percentage of drug exposure per day of hospitalization, which could add value to the comparisons between gestational age groups. We can assume that most neonates who received a continuous infusion of opioids, sedatives, or paralytics were on mechanical ventilation and that single doses were used for procedural pain (including intubation), but we cannot clearly describe prescription practices according to clinical context (Borenstein-Levin et al., 2017). Second, this database reported prescriptions but not bedside treatment administrations. Thus, some therapies might not have been prescribed but instead administered at bedside, such as loading doses or boluses. Conversely, some prescribed medications might not have been administered due to decision changes. Third, we had no information on outcomes or adverse effects. This study nonetheless provides an exhaustive picture of actual prescription practices, because all validated prescriptions were prospectively recorded for all patients in each NICU.

## Conclusion

In a large cohort of neonates hospitalized in 30 French NICUs over a 6-year period, 44.8% of patients were prescribed at least one analgesic, sedative, anesthetic, or paralytic agent: 17.8% opioids, 9.8% sedatives, 8.5% anesthetics, and 1% paralytics. These agents, apart from acetaminophen, were mainly prescribed combined with one another. Preterm neonates, and especially those born before 28 weeks were far more exposed—in terms of frequency, cumulative dose, and duration of prescriptions—than term neonates. Prescribed doses varied by GA for most substances. This pharmacoepidemiologic study, presenting an overview of prescription practices to assess the current use of these drugs, could help to develop realistic strategies to prevent or relieve painful and stressful situations in neonates. Dose-finding studies taking into account GA and long-term safety studies taking into account received cumulative doses are needed to develop individualized, safe, and effective strategies for neonatal sedation and analgesia.

## Data Availability

The datasets presented in this article are not readily available because the procedures carried out with the French data privacy authority (CNIL, Commission nationale de l'informatique et des libertés) do not provide for the transmission of the database. Requests to access the datasets should be directed to manon.tauzin@chicreteil.fr.
